# Evaluation of publication type tagging as a strategy to screen randomized controlled trial articles in preparing systematic reviews

**DOI:** 10.1093/jamiaopen/ooac015

**Published:** 2022-03-30

**Authors:** Jodi Schneider, Linh Hoang, Yogeshwar Kansara, Aaron M Cohen, Neil R Smalheiser

**Affiliations:** 1 School of Information Sciences, University of Illinois Urbana-Champaign, Champaign, Illinois, USA; 2 Department of Medical Informatics and Clinical Epidemiology (DMICE), School of Medicine, Oregon Health & Science University, Portland, Oregon, USA; 3 Department of Psychiatry, College of Medicine, University of Illinois Chicago, Chicago, Illinois, USA

**Keywords:** RCT Tagger, systematic review automation, randomized controlled trials, information retrieval

## Abstract

**Objectives:**

To produce a systematic review (SR), reviewers typically screen thousands of titles and abstracts of articles manually to find a small number which are read in full text to find relevant articles included in the final SR. Here, we evaluate a proposed automated probabilistic publication type screening strategy applied to the randomized controlled trial (RCT) articles (i.e., those which present clinical outcome results of RCT studies) included in a corpus of previously published Cochrane reviews.

**Materials and Methods:**

We selected a random subset of 558 published Cochrane reviews that specified RCT study only inclusion criteria, containing 7113 included articles which could be matched to PubMed identifiers. These were processed by our automated RCT Tagger tool to estimate the probability that each article reports clinical outcomes of a RCT.

**Results:**

Removing articles with low predictive scores *P* < 0.01 eliminated 288 included articles, of which only 22 were actually typical RCT articles, and only 18 were actually typical RCT articles that MEDLINE indexed as such. Based on our sample set, this screening strategy led to fewer than 0.05 relevant RCT articles being missed on average per Cochrane SR.

**Discussion:**

This scenario, based on real SRs, demonstrates that automated tagging can identify RCT articles accurately while maintaining very high recall. However, we also found that even SRs whose inclusion criteria are restricted to RCT studies include not only clinical outcome articles per se, but a variety of ancillary article types as well.

**Conclusions:**

This encourages further studies learning how best to incorporate automated tagging of additional publication types into SR triage workflows.

## Lay Summary

In medical research, treatments are compared using randomized controlled trials (RCTs). To identify safe and effective treatments, systematic reviews are carried out which identify and analyze articles that present the results from multiple trials on the same question. We have previously created a tool called RCT Tagger, which identifies RCT articles automatically. The present paper verifies that the RCT Tagger would have identified nearly all relevant articles when applied to previously published systematic reviews. This suggests that people writing systematic reviews should consider automated publication type tagging as part of their screening for relevant articles.

## INTRODUCTION

Systematic reviews (SRs) are a type of literature review designed to provide the best evidence on a given question.^1^ The current best practices for writing SRs require a great amount of manual time and effort^2^ to identify comprehensively all relevant publications for evidence synthesis. A worldwide effort has begun to create automated tools to assist in both the retrieval of relevant articles and the extraction of information from these articles.^3^^,^^4^ Most of the retrieval tools have focused on identifying articles that are relevant based on topical, textual, or patient inclusion criteria.^5–13^ However, an article’s publication type and study design characteristics are also important aspects of its relevance for inclusion. Randomized controlled trials (RCTs) are considered the gold standard for knowledge about the effects of medical treatments,^14^ and finding reports of RCTs in a list of search results is critical for selecting the papers to be summarized in SRs.^15–17^ Recently, we and others have developed automated and semiautomated publication type taggers to identify articles that present clinical outcomes of RCTs.^9^^,^^18^^,^^19^ Publication type tagging has been proposed to potentially contribute to the initial screening of articles during triage,^9^^,^^18^^,^^20^ but has not yet been widely implemented.

“RCT Tagger,” a machine learning-based model, which estimates the probability that a given biomedical article reports the clinical outcome of a RCT,^18^ achieves high accuracy (AUC ≥ 0.984) when evaluated with MEDLINE’s “Randomized Controlled Trial” Publication Type^21^ and EMBASE citations as gold standards.^19^ However, further considerations and evaluations are needed in order to implement RCT Tagger as part of the workflow of writing a SR. RCT Tagger might be implemented in several different modes, for example, a filter-in strategy in which only high-scoring articles are retained, or a filter-out strategy in which low-scoring articles are thrown out. Here, we decided to test a filter-out strategy in which any article having a predicted probability score <0.01 is discarded. Theoretically this threshold should discard fewer than 1% of relevant articles (achieving >99% recall); however, it is important to assess this screening strategy in a more stringent and pertinent manner using a realistic scenario using published Cochrane SRs. These SRs give an explicit list of the articles that were manually reviewed, deemed relevant, and finally included for evidence synthesis. Since a typical SR may only contain 5–50 included articles, mistakenly filtering out even one included article may be considered unacceptable.

## OBJECTIVES

We ask whether filtering out articles having RCT Tagger predictive probability scores < 0.01 retains at least 99% of the relevant RCT articles included in a corpus of previously published Cochrane reviews.

In terms of consistent terminology, we must distinguish 3 concepts related to RCTs: the trials/studies themselves, the RCT articles describing trial outcomes, and ancillary articles linked to trials such as reviews, protocols, reanalyses of data, and embedded studies. As Cochrane notes, “Systematic reviews have studies, rather than reports, as the unit of interest, and so multiple reports of the same study need to be identified and linked together before or after data extraction…a study can be reported in multiple journal articles, each focusing on some aspect of the study (e.g. design, main results, and other results).”^22^ Cochrane describes a RCT as “An experiment in which 2 or more interventions, possibly including a control intervention or no intervention, are compared by being randomly allocated to participants. In most trials one intervention is assigned to each individual but sometimes assignment is to defined groups of individuals (for example, in a household) or interventions are assigned within individuals (for example, in different orders or to different parts of the body).”^23^ We defined RCT articles in our previous research^18^; here, we simplify the definition to “An RCT article reports the primary or secondary outcomes of an RCT study.” In the rest of the paper, we will distinguish trials (RCT studies), reports describing the trial outcomes (RCT articles), and ancillary articles; we will also refer to our model (RCT Tagger).

## MATERIALS AND METHODS

We constructed a corpus consisting of a large random sample of Cochrane reviews. For convenience, we only considered articles that are indexed in PubMed, since all articles in PubMed have been indexed with RCT Tagger prediction scores and are incremented weekly.^24^ (Articles not indexed in PubMed can also be given prediction scores but we have not comprehensively tagged other bibliographic databases as yet.) Also, we only analyzed Cochrane reviews whose inclusion criteria focused solely on RCT studies, because in these cases, the great majority of included articles were RCT articles. Note that a given RCT study may generate many diverse types of published articles (e.g., secondary analysis of data, genome-wide association studies of human subjects, embedded case-control analyses, etc.), which are not themselves RCT articles (i.e., reports of the primary clinical outcomes of the trial).

**Figure 1.**
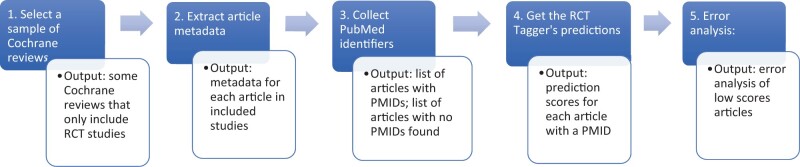
Main steps and outputs of our evaluation process.

Our process was comprised of 4 steps, as shown in Figure 1: (1) Select a random sample of Cochrane reviews; (2) Extract article metadata for each article included in the sampled reviews; (3) Collect PubMed identifiers (PMIDs) for each article; and (4) Obtain the RCT Tagger prediction scores. Each step is described in further detail below.

### Select a sample of Cochrane reviews

We selected Cochrane reviews from within a XML-formatted dataset, received directly from Cochrane, consisting of 7158 reviews published from 2008 through January 3, 2018 by 52 different Cochrane groups in 8 Cochrane group networks.^25^ These were stratified by publication year and Cochrane group network, and we selected 15% randomly from each bin. Of these, we included only reviews whose inclusion criteria was restricted to RCT studies based on our manual annotation, and filtered out empty reviews (i.e., those that contained zero included studies).

### Extract article metadata

We extracted metadata about each article in an included study from a sampled review. To do this, we ran a program to process the XML files for each review, which extracted 3 levels of metadata: Review, Study, and Article as shown in Table 1.

**Table 1.** List of metadata extracted from XML files for each review

#	Field name	Level of metadata	Example metadata
1	Review name	Review	CD007474 v. 6.0 Risperidone dose for schizophrenia.rm5
2	Study name	Study	Marder 1994
3	Study ID	Study	STD-Marder-1994
4	Title	Article	Successful therapy with risperidone in schizophrenic negative syndrome
5	Alternative title	Article	Schizophrenes Negativsyndrom. Risperidon Erfolgreich
6	Authors	Article	Blaeser-Kiel G
7	Type of article	Article	JOURNAL_ARTICLE
8	Published journal	Article	TW Neurologie Psychiatrie
9	Year	Article	1994
10	Volume	Article	8
11	Page	Article	614-5
12	Reference ID	Article	1994342404
13	Reference ID type	Article	EMBASE
14	Reference ID other type	Article	CRSREF

### Collect PMIDs for articles

To collect PMIDs for the articles, the PubMed API^26^ was queried for PMIDs matching each article’s metadata. First, we used the ECitMatch API^27^ because it determines exact matches between article metadata and a PMID. For each article, we input to ECitMatch its publication year, journal, volume, and page numbers.

As a second pass, for articles not matched by the ECitMatch API, we used the ESearch API^27^ because it returns a list of PMIDs as results of a single text query. Input was the title, the first author, and the publication year. Since the API could return multiple potential matched PMIDs or no matched PMIDs, the second-round API results were manually validated by comparing to the original metadata from the source Cochrane review. This resulted in 2 lists: a list of unmatched articles and a list of PMIDs for articles included in studies in our sample of reviews and available in PubMed. For each matched PMID, we also retrieved the article’s title, abstract, and MEDLINE Publication Types.

As a third pass, for each article with a matched PMID, we compared its title and abstract from the original Cochrane Review against the match retrieved from the PubMed API. This resulted in 2 lists: a list of articles that had a PMID mapping error (which we excluded); and a list of articles with confirmed PMID matches.

### Get RCT Tagger prediction scores

We queried the RCT Tagger on the PMIDs retrieved using the public query interface (http://arrowsmith.psych.uic.edu/cgi-bin/arrowsmith_uic/RCT_Tagger.cgi).

## RESULTS

**Figure 2.**
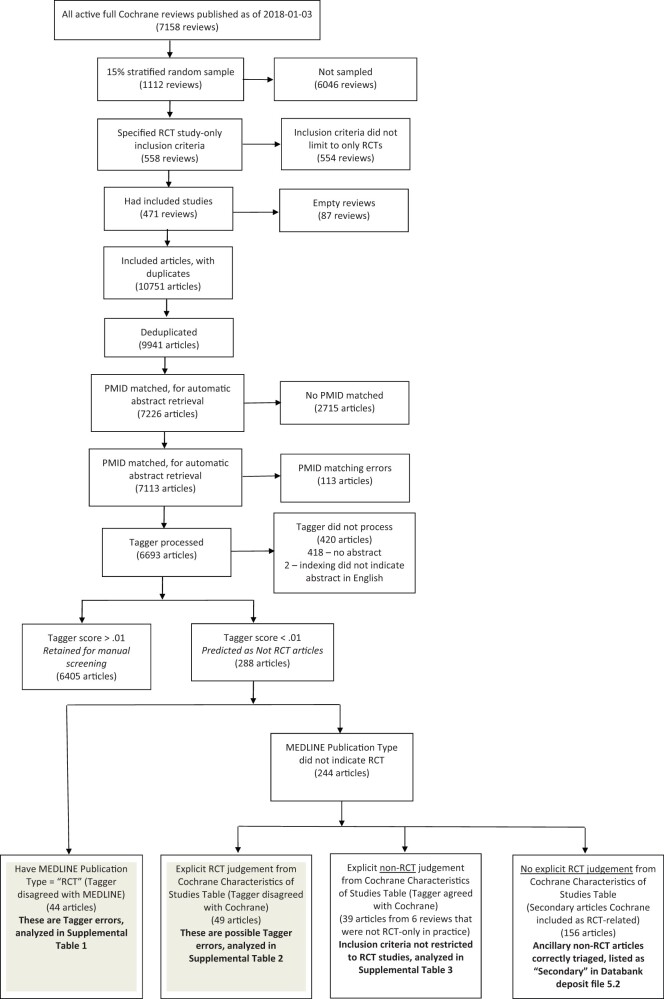
Our evaluation strategy started with a 15% stratified sample and ultimately analyzed 6693 Tagger processed articles from 471 Cochrane reviews. Each article considered in the analysis ended in one of 5 outcomes: retained for manual screening (6405 articles); Tagger error (44 articles); possible Tagger error (49 articles); explicit nonRCT judgment from Cochrane Characteristics of Studies Table (39 articles from 6 reviews); or explicit nonRCT judgment from Cochrane Characteristics of Studies Table (156 articles).

Figure 2 shows our evaluation strategy. Briefly, starting with a 15% stratified sample, we ultimately analyzed 6693 Tagger processed articles from 471 Cochrane reviews. Each article considered in the analysis ended in 1 of 5 outcomes: retained for manual screening (6405 articles); Tagger error (44 articles); possible Tagger error (49 articles); explicit nonRCT judgment from Cochrane Characteristics of Studies Table (39 articles from 6 reviews); or explicit nonRCT judgment from Cochrane Characteristics of Studies Table (156 articles). We now describe our process and error analysis in further detail.

From the full set of 7158 Cochrane reviews, our 15% stratified sample yielded 1112 reviews, and we retained the 558 reviews that we annotated as having RCT-only inclusion criteria. Our final set of reviews consisted of the 471 reviews that had at least 1 included study. After deduplicating articles included in multiple reviews, we attempted to match 9941 articles to PMIDs. Of the 7226 articles matched to PMIDs, we removed 113 (1.5%) articles that had PMID mapping errors. Of the remaining 7113 articles with confirmed PMIDs matches, 6693 articles received estimated probability scores from RCT Tagger. The other 420 articles either had no abstract in PubMed, or the full-text was not in English and the article was not indexed as having an English abstract in the Publication Type metadata field. Parenthetically, although it is rare for an RCT article representing a primary report of a clinical trial outcome to be published without an abstract, this enumeration suggests that articles lacking abstracts should not be automatically discarded during literature screening.

Among the 6693 articles scored by RCT Tagger, 288 articles had predictive probability scores below 0.01. We conducted an error analysis of these low-scoring articles. According to MEDLINE Publication Type, only 44 of these low-scoring articles were indexed as RCT articles, and the remaining 244 of these low-scoring articles were not indexed as RCT articles.

For the 44 low-scoring articles that were indexed as RCT articles according to MEDLINE Publication Type, we manually examined the full text of and found that actually only 18 of the 44 articles were typical RCT articles (see Supplementary Table S1). The others were borderline cases (e.g., cluster randomization, blinding not mentioned) or appeared to be frankly not RCT articles at all (e.g., posthoc analysis, nested case control study, or data reanalysis).

For the 244 low-scoring articles not MEDLINE-indexed as RCTs, only 49 primary articles had been explicitly judged to be RCT articles by Cochrane. We found 8 main reasons that Tagger missed them: Abstract field empty in XML, Abstract lacks detail, Comparative study with randomization not made explicit in abstract, Design, Diagnostic test accuracy, Technical language, Topic atypical, Typical RCT (Supplementary Table S2). An additional 39 primary articles from 6 Cochrane’s SR’s had been explicitly judged by Cochrane to be nonRCT articles (e.g., quasi-randomized trials, comparative studies, community-based trials, surveys) according to Cochrane’s Characteristics of Studies table; rereading those SR’s inclusion criteria, we determined that we had misclassified 3 SRs as “RCT only” and that the Cochrane authors had expanded inclusion criteria in the other 3 SRs (see Supplementary Table S3). The remaining 156 low-scoring articles were ancillary articles which did not have explicit study-design judgments recorded in the Cochrane SR’s Characteristics of Studies table; Cochrane includes ancillary articles as companions to some primary RCT article.

Thus, using RCT Tagger for filtering out articles with scores < 0.01 retained (6693 – (44 + 49))/6693 = 98.6% of the RCT articles included in the corpus of 471 Cochrane SRs. Filtering by using RCT Tagger along with MEDLINE would have retained (6693 – 49)/6693 = 99.27% of the RCT articles.

If one only considers articles that our expert review confirmed were typical RCT articles (see Supplementary Material), the proportion is (6693 – 22)/6693 = 99.67% of the included articles. Stated otherwise, our proposed screening strategy would on average lead to only 22 articles/471 Cochrane reviews = 0.047 RCT articles being mistakenly discarded per Cochrane SR.

## DISCUSSION

In the present paper, we have demonstrated that an automated probabilistic publication type screening strategy, specifically, filtering out articles having RCT Tagger predictive probability scores < 0.01, retains well over 98% of the relevant RCT articles included in a corpus of previously published Cochrane reviews. Stated another way, fewer than 0.05 RCT articles per Cochrane SR would be mistakenly discarded using this strategy.

What might this mean for a real-world application of RCT Tagger? Applying the tool to the initial set of articles retrieved from database queries, one would filter out articles with very low predictive scores (<0.01) prior to giving to SR teams for manual triage. In our earlier study, we estimated that ∼85% of articles would be removed by RCT Tagger using a threshold of 0.1.^18^ It was not possible for us to calculate work savings precisely in the present study, since unfortunately, few if any published Cochrane reviews provide an explicit list of the initially retrieved articles used for manual screening. The queries that were provided in our corpus are impossible to rerun exactly because they vary in terms of the databases and search engines involved, which themselves change over time. However, for 4 randomly selected Cochrane reviews within our dataset, we attempted to reconstruct their initial PubMed queries as closely as possible. Applying RCT Tagger to remove articles with scores below 0.01, we found that an average of 64% of the initially retrieved articles were removed. This is admittedly a rough estimate but suggests that publication type screening does offer the promise of saving substantial effort in manual triage, and encourages prospective studies of SRs (where the initial set of retrieved articles is known exactly) to calculate work savings more robustly.

Ultimately, the contribution of automated publication type tagging needs to be evaluated in the context of, and in combination with, other machine learning approaches to relevance ranking such as RobotReviewer, RobotSearch, Abstrackr, SWIFT-Active Screener, and SWIFT-Review, SRA-Helper, and DistillerSR^6–11^ as well as other manual strategies that systematic reviewers routinely use to find relevant literature (e.g. following citation trails, articles written by specific authors, or publications linked to registered trials). The optimal threshold for RCT Tagger, and the overall work savings obtained, will be a function not only of the tagger itself, but of the entire workflow involving all automated tools.

Our study has certain limitations: The evaluation was restricted to articles that we could match to PMIDs, i.e. indexed in PubMed. In addition, a small number (∼410 of 7113) of articles included in the SRs had also been included in the training data used in modeling RCT Tagger^18^; however, this is unlikely to impact the results.

## CONCLUSIONS

The present study is proof-of-principle involving a single (albeit dominant) publication type, the RCT. However, as we found, even SRs that are restricted to RCT studies include not only RCT articles but a variety of ancillary articles as well. And, many SRs include a variety of study designs in their inclusion criteria. Therefore, it will be necessary to carry out automated screening for multiple publication types and study designs, such as cohort studies, case control studies, and cross-sectional studies, which are also relevant for inclusion in many SRs. We have created such a series of taggers^28^ and plan to evaluate their utility for SR triage in the near future.

## FUNDING

This study was funded by a grant from the National Library of Medicine, “Text Mining Pipeline to Accelerate Systematic Reviews in Evidence-based Medicine” (R01LM010817). The funding agency had no role in the preparation, review, or approval of the manuscript. The opinions, results, and conclusions reported in this paper are those of the authors and are independent of the funding source.

## AUTHOR CONTRIBUTIONS

CRediT Roles: Conceptualization—NRS, AMC, JS. Data curation—LH, YK, JS, Xiaoru Dong, Randi Proescholdt, and Jingyi Xie. Formal analysis—Funding acquisition—NRS, AMC, JS. Investigation—LH, YK, JS. Methodology—NRS, AMC, JS, LH. Project administration—JS. Resources—NRS, AMC, JS. Software—LH, YK, https://github.com/infoqualitylab/Tagger_Evaluation. Supervision—NRS, AMC, JS. Visualization—LH, YK. Writing—original draft—LH, JS. Writing—review, and editing—NRS (lead), JS, AMC, YK, JS, LH.

## SUPPLEMENTARY MATERIAL

Supplementary material is available at *JAMIA Open* online.

## Supplementary Material

ooac015_Supplementary_Data
