# Supplementary material for: Evaluation of publication type tagging as a strategy to screen randomized controlled trial articles in preparing systematic reviews
Source: JAMIA Open. 2022 Mar 30;5(1):ooac015. doi: 10.1093/jamiaopen/ooac015 (PMC9097760; doi:10.1093/jamiaopen/ooac015)
Supplement: ooac015_Supplementary_Data [file ooac015_supplementary_data.docx]

# **SUPPLEMENT**

Table S1 – Manual review and categorization of 44 articles in included studies given scores <0.01 by RCT Tagger but MEDLINE indexed as RCTs.

| **Category** | **Number of articles** | **Example full text excerpt supporting the category judgment** |
| --- | --- | --- |
| Open trial | 3 | “no blinded, open-trial design with no placebo control group” & “Participants were all randomly assigned to Sertraline, Paroxetine, and Venlafaxine conditions.” (PMID 11534876) |
| Posthoc analysis | 1 | “This was a post-hoc analysis of data collected for the WHI clinical trials.” (PMID 20354566) |
| Blinding not mentioned | 1 | “We treated 1622 patients due to chronic venous insufficiency (CHVI) in the past 10 years (1991-2000). In this prospective randomized study there were 3 groups of patients” (PMID 12839217) |
| RCT related | 12 | “matched case–control study nested within the prospective Physicians’ Health Study” (PMID12376472) |
| Reanalysis | 3 | “In this study, we examined the relative strength of association of measures of community functioning, as assessed by the Quality of Life Scale and self-reported days of employment, with measures of both neurocognition and symptoms. The data were from the Clinical Antipsychotic Trials of Intervention Effectiveness (CATIE) schizophrenia study, a large randomized, controlled trial funded by the National Institute of Mental Health and designed to compare outcomes of antipsychotic medications” (PMID 18450928) |
| Unit of randomization | 2 | “The effectiveness evaluation of Safer Choices was a randomized trial involving 10 schools in northern California and 10 schools in southeast Texas.” (PMID 11030854) |
| Verified as RCT by manual reading of full text | 18 | “The 96 subjects who completed the initial assessment were randomly allocated to one of four different training programmes designed to characterize common components of published tinnitus management programs…” (PMID 10219721) |
| Not RCT | 2 | “This is an intervention study with historical control” “a control group was not desired.” (PMID 21986721) |
| Full-text not in English | 2 | (non-English content) (PMID 786313) |

Two graduate students (LH, YK) read the full text of each article and did the manual RCT publication type annotations following written criteria; JS checked their spreadsheet (File5_ErrorAnalysis.xlsx; Kansara, Yogeshwar; Hoang, Linh; Schneider, Jodi (2019): Error Analysis. University of Illinois at Urbana-Champaign. https://doi.org/10.13012/B2IDB-3407079_V3) and checked article PDFs when questions arose. Of the 44 articles that received MEDLINE RCT Publication Type indexing, shown are the article designs as decided by the annotators, the number of articles in each category, and extracted full-text that supported our judgments. Only 18 of the 44 articles were verified as typical RCT articles.

Table S2 – Manual review and categorization of 49 articles in included studies given scores <0.01 by RCT Tagger, not indexed as RCTs by MEDLINE, but explicitly judged to be RCTs by Cochrane

| **Category** | **Number of articles** | **Example abstract supporting the category judgment** |
| --- | --- | --- |
| **Technical language** | **14** | PMID 2961624: “Serum concentrations of CA-125 were determined in association with 6-month medical (n = 48) or surgical and medical therapy (n = 40) of endometriosis. The concentration of CA-125 was significantly higher in stages III + IV (66.6 +/- 22.0 [standard deviation] U/ml) than in stage I (20.9 +/- 2.3 U/ml) or II (28.4 +/- 2.8 U/ml); in stage II, the concentration was higher than in stage I. Surgical elimination of endometriosis significantly decreased the level of CA-125, as did danazol, but not medroxyprogesterone acetate (MPA), although these drugs were equal in clinical efficacy. The CA-125 changes during hormonal treatment did not correlate with the clinical response. Postoperatively, CA-125 responses to danazol, MPA, or placebo did not differ significantly from each other. During the 6-month follow-up after medication, the CA-125 concentrations tended to increase, especially in danazol-treated women. The determination of CA-125 is useful in estimating the extent of the disease, but it is less valuable in monitoring the treatment effect. The ability of danazol to suppress CA-125 expression emphasizes the specific properties of this drug.” |
| **Abstract lacks detail** | **8** | PMID 949108: “The hypnotic agent etomidate was added by infusion to a fentanyl-N2O-O2-anaesthesia for patients (n=15) requiring open heart surgery. The purpose of this procedure was to avoid awareness during operation (mean duration 187 min). The average amount of etomidate infused was 2.55 mg/kg, which seemed to be an overdose. The fentanyl consumption was slightly reduced as compared with a control group. No patients complained of awareness. The anaesthetic method studied is not thought to be superior to a high-dose fentanyl anaesthesia.” |
| **Design** | **7** | PMID 6581062: Nine adult asthmatics took part in a cumulative dose response comparison of fenoterol (Berotec) inhalation powder and fenoterol metered dose inhaler. The study was carried out as a double-blind investigation using a double-dummy technique. No significant difference was observed in the lung function, in tremor or pulse rate on comparison of the two modes of administration. It is concluded that fenoterol inhalation powder is an effective and freon-free alternative to the metered dose inhaler. |
| **Comparative study with randomization not made explicit** | **5** | PMID 3787305: “The iron status of voluntary and professional male donor groups was investigated. The study indicated that serum ferritin level was lower significantly in those who donated three times per year compared to the first time donors (p less than 0.05) in voluntary donor group. Similar results were observed among the professional donors. Whereas haemoglobin, haematocrit and transferrin saturation were not altered by donating blood in both groups. It also showed that, in general, the body iron stores of professional donors were lower to those of voluntary donors. Supplementation with iron over a period of three months produced a rise in serum ferritin levels and the percentage prevalence of iron depleted subjects decreased from 23.6% to 6.4%. Haemoglobin, haematocrit and transferrin saturation levels also improved.” |
| **Abstract field empty in XML** | **5** | PMID 2830038, PubMed “No abstract available”; article not in English; title [Research on the "anti-aging" effect of breathing exercises (qigong)]  PMID: 12317444, PubMed XML uses Other abstract; title A randomised comparative study of Triquilar versus Marvelon: the Malaysian experience |
| **Topic atypical** | **4** | PMID 1817616: “This paper reports the findings of a 20-week social adjustment enhancement curriculum for boys aged 8-12. The curriculum was designed to address three areas hypothesized to be deficient in persons with HFA, AS, and PDDNOS: emotion recognition and understanding; theory of mind; and executive functions/real life type problem solving. Parents attended a semi-structured concurrent psychoeducational training meeting during children's sessions. Statistically significant improvements in facial expression recognition, and problem solving were reported for intervention group children compared to waiting list control group children. For the intervention group (the only group for whom data were available), older and less cognitively able boy's scores on a depression inventory decreased significantly more than younger children's. Mother's depression scores tended to decrease and there were significant reductions in child problem behaviors reported. Results are discussed in the context of individual differences in participant cognitive levels and profiles, symptom severity, and affect-related variables.” |
| **Typical RCT** | **4** | PMID 3133710: “We are performing a double-blind trial with inward psychiatric patients. The indication for our psychotropic or psychotherapeutic intervention is mainly severe depression (= major depressive disorders DSM III). For a 3-week trial course trazodone (400 mg daily), amitriptyline (150 mg/die) or placebo capsules were given at random. All patients received the same type of cognitive behaviour therapy. The test battery consists of CGI, BPRS, HAMD, HAMA and AMDP; adverse drug reactions are documented as "free reports" (= freier Nebenwirkungsbericht). The interim results (until March 1987) will be presented. Our investigation indicates that it is probable that the trazodone treatment we used is equivalent to corresponding amitriptyline treatment.” |
| **Diagnostic test accuracy** | **3** | The performance of a new ectocervical brush sampler--the Cervex--was compared with the Ayre spatula in 280 paired cervical smears. The Cervex smears were superior in quality of spread, transformation zone sampling in all degrees of cervical patency and in detection of histologically proven epithelial abnormalities, with a false negative rate of 10.9% compared with 20% for the Ayre. Improvement in predictive value was noted in atrophic samples, with increased cellularity and transformation zone representation. Difficulty has been encountered in obtaining adequate samples from the older woman and from those with iatrogenic scarring of the cervix. Although two-sampler techniques may be used, submission of high quality pan-cervical material from a single sampler onto one slide is economically and organizationally attractive. The Cervex seems capable of producing such samples and deserves further evaluation for routine screening. (PMID 2102356) |

The table shows the reasons for Tagger’s error according to one annotator (JS, by reading abstracts), the number of articles in each category, and extracted abstract that supported our judgments. Only 4 of the 49 abstracts described typical RCT articles. Supporting data in Kansara, Yogeshwar; Hoang, Linh; Schneider, Jodi (2022): Error Analysis. University of Illinois at Urbana-Champaign. https://doi.org/10.13012/B2IDB-3407079_V3

Table S3 – Manual review and categorization of 6 Cochrane reviews contributing 39 articles explicitly judged by Cochrane’s Characteristics of Studies tables to be NOT RCTs (given scores <0.01 by RCT Tagger, not indexed as RCTs by MEDLINE)

| **Review Name** | **Review Inclusion Criteria** | **Number of articles contributed to the 244 low-scoring articles** | **Article study design(s) according to Cochrane Characteristics of Studies Table** |
| --- | --- | --- | --- |
| CD000110 v. 7.0 Hospitalisation and bed rest for multiple pregnancy.rm5 | Inclusion is wider than specified criteria | 1 | Quasi-random trial |
| CD001036 v. 11.0 Collection devices for obtaining cervical cytology samples.rm5 | Inclusion is wider than specified criteria | 13 | Comparative study, Quasi-random trial |
| CD003628 v. 6.0 Artificial and bioartificial support systems for liver failure.rm5 | Not RCT only inclusion criteria | 1 | Non-randomized (included for exploratory analysis only) |
| CD004445 v. 8.0 The 'WHO Safe Communities' model for the prevention of injury in whole populations.rm5 | Not RCT only inclusion criteria | 17 | Non-randomized community controlled study |
| CD004446 v. 10.0 Emergency ultrasound-based algorithms for diagnosing blunt abdominal trauma.rm5 | Inclusion is wider than specified criteria | 2 | Quasi-random trial |
| MR000021 v. 4.0 Incentives and disincentives to participation by clinicians in randomised controlled trials.rm5 | Not RCT only inclusion criteria | 5 | Interviews, RCT-related survey, Survey |

The table shows the review name, the nature of the error according to one annotator’s judgement (JS) of the review inclusion criteria, the number of articles involved, and their article study designs according to the Cochrane Characteristics of Studies Table. Supporting data (including review inclusion criteria) in Kansara, Yogeshwar; Hoang, Linh; Schneider, Jodi (2022): Error Analysis. University of Illinois at Urbana-Champaign. https://doi.org/10.13012/B2IDB-3407079_V3
